# Probiotic Potentials and Protective Effects of *Ligilactobacillus animalis* LA-1 Against High-Fat Diet-Induced Obesity in Mice

**DOI:** 10.3390/nu17142346

**Published:** 2025-07-17

**Authors:** Qingya Wang, Yuyin Huang, Kun Meng, Haiou Zhang, Yunsheng Han, Rui Zhang, Xiling Han, Guohua Liu, Hongying Cai, Peilong Yang

**Affiliations:** 1 Key Laboratory of Feed Biotechnology of Ministry of Agriculture and Rural Affairs, Institute of Feed Research, Chinese Academy of Agricultural Sciences, Beijing 100081, China; 18238693320@163.com (Q.W.); yuyinhuang99@163.com (Y.H.); mengkun@caas.cn (K.M.); zhanghaiou@caas.cn (H.Z.); hanyunsheng@caas.cn (Y.H.); 17789363681@163.com (X.H.); liuguohua@caas.cn (G.L.); 2 Key Laboratory of Yunnan for Biomass Energy and Biotechnology of Environment, Yunnan Normal University, Kunming 650500, China; ruizhang@ynnu.edu.cn

**Keywords:** *Ligilactobacillus animalis* LA-1, probiotic activities, high-fat diet, obesity, gut microbiota, SCFAs, amino acid metabolism

## Abstract

**Background/Objectives**: Obesity is increasingly recognized as a global health concern due to its association with metabolic disorders and gut microbiota dysbiosis. While probiotics offer promise in regulating gut microbiota and improving host metabolism, strain-specific effects remain underexplored, particularly for canine-derived probiotics. This study aimed to isolate and characterize a novel probiotic strain, *Ligilactobacillus animalis* LA-1, and evaluate its anti-obesity effects and underlying mechanisms using a high-fat diet (HFD)-induced obese mouse model. **Methods**: LA-1 was isolated from the feces of a healthy dog and assessed for probiotic potential in vitro, including gastrointestinal tolerance, bile salt hydrolase activity, cholesterol-lowering capacity, and fatty acid absorption. Male C57BL/6J mice were fed either a standard chow diet or an HFD for 16 weeks, with HFD mice receiving oral LA-1 supplementation (2 × 10^9^ CFU/day). Multi-omics analyses, including 16S rRNA gene sequencing, short-chain fatty acid (SCFA) quantification, and untargeted liver metabolomics, were employed to investigate the effects of LA-1 on gut microbiota composition, metabolic pathways, and obesity-related phenotypes. **Results**: LA-1 supplementation significantly alleviated HFD-induced weight gain, hepatic lipid accumulation, and adipose tissue hypertrophy, without affecting food intake. It improved serum lipid profiles, reduced liver injury markers, and partially restored gut microbiota composition, decreasing the Firmicutes/Bacteroidetes ratio and enriching SCFA-producing genera. Total SCFA levels, particularly acetate, propionate, and butyrate, increased following LA-1 treatment. Liver metabolomics revealed that LA-1 modulated pathways involved in lipid and amino acid metabolism, resulting in decreased levels of acetyl-CoA, triglycerides, and bile acids. **Conclusions**: *L. animalis* LA-1 exerts anti-obesity effects via gut microbiota modulation, enhanced SCFA production, and hepatic metabolic reprogramming. These findings highlight its potential as a targeted probiotic intervention for obesity and metabolic disorders.

## 1. Introduction

Obesity is a growing global health concern and a major risk factor for chronic diseases, including type 2 diabetes, cardiovascular disease, and non-alcoholic fatty liver disease [1,2,3]. In recent years, its prevalence has also sharply increased among pets [4,5], with 40–60% of dogs affected [6]. Beyond appearance, obesity in animals is associated with insulin resistance, dyslipidemia, hepatic steatosis, and immune dysfunction [7,8,9]. Moreover, obesity significantly shortens the lifespan of pets, reduces their quality of life, and imposes emotional and financial burdens on pet owners [9]. Although conventional pharmacological treatments can improve certain clinical indicators, they are often limited by adverse side effects, poor compliance, and uncertain long-term efficacy [10,11]. These trends highlight the urgent need for effective, safe, and sustainable interventions targeting obesity in both humans and companion animals.

The gut microbiota plays a central role in host metabolism by regulating nutrient absorption, fat storage, and hepatic lipid homeostasis [12,13,14]. Dysbiosis has been closely associated with obesity, insulin resistance, and type 2 diabetes [15,16]. Microbial metabolites such as short-chain fatty acids (SCFAs) mediate host-microbiota interactions, impacting lipid synthesis, glucose homeostasis, and energy balance [17,18]. Therefore, restoring gut microbial homeostasis is considered a promising approach for obesity intervention.

Probiotic modulation of gut microbiota has shown promise in improving host metabolism [19]. Traditional strains like *Lactobacillus* and *Bifidobacterium* can alleviate metabolic disorders through various mechanisms [20,21]. For instance, *Lactobacillus rhamnosus* GG can alleviate oxidative stress and inflammation in rats with non-alcoholic fatty liver disease [22]. Both live and heat-inactivated *Bifidobacterium animalis* J-12 have been reported to relieve oral ulcers in golden hamsters by modulating gut microbiota [23]. *Lactiplantibacillus plantarum* FRT4 has been found to prevent fatty liver hemorrhagic syndrome by regulating gut microbiota and the FOXO/TLR-4/NF-κB signaling pathway [24]. However, probiotic effects are highly strain-specific, and even closely related strains may differ in their metabolic impacts [25,26]. Most current studies focus on food- or human-derived strains, while limited attention has been given to probiotics from companion animals [27,28,29]. This limits targeted applications in pet nutrition and disease intervention. Notably, canine-derived *Lactobacillus* strains may offer host-adapted advantages within the specific gut environment of dogs, providing a more effective approach to modulating metabolism and obesity in pets [30,31,32]. *Ligilactobacillus animalis* is a probiotic commonly found in the gastrointestinal tract of animals. It exhibits strong antimicrobial activity and anti-inflammatory properties and contributes to the protection of the intestinal barrier [33,34]. However, its functional mechanisms in models of obesity-related diseases have not been systematically elucidated, and its potential as a functional ingredient in pet nutrition remains to be fully explored.

In this study, a canine-derived *Lactobacillus animalis* strain LA-1 with probiotic potential was isolated and evaluated for its probiotic properties, safety, and anti-obesity effects in vitro. Moreover, we established a high-fat diet (HFD)-induced obese mouse model and conducted a comprehensive evaluation using multi-omics approaches, including 16S rRNA gene sequencing, SCFA quantification, and untargeted hepatic metabolomics. Murine models are widely accepted for preclinical probiotic evaluation due to their well-characterized metabolic profiles and suitability for mechanistic investigation. This cross-species approach enables translational insights and provides a foundational basis for future applications in companion animals. This study systematically evaluates canine-derived *L. animalis* in obesity intervention, offering novel insights and microbial targets for pet-adapted probiotic development and microecological management of metabolic disorders.

## 2. Materials and Methods

### 2.1. Preparations of L. animalis LA-1 and Pathogenic Strains

LA-1 was isolated from the feces of a healthy dog and deposited in the Chinese General Microbiological Culture Collection Center (CGMCC) with the accession number CGMCC No. 32098 after identification. Before the experiment, LA-1 was revived on MRS agar at 37 °C for 48 h. A single, well-isolated colony was then inoculated into MRS broth and cultured at 37 °C for 24 h. Subsequently, a 2% inoculum was transferred into 50 mL fresh MRS broth and incubated under the same conditions for another 24 h. The cultured cells were washed twice with 0.01 M phosphate-buffered saline (PBS, pH 7.2), and the cell density was adjusted to 1 × 10^9^ CFU/mL in PBS for further experiments.

Pathogenic strains (*Staphylococcus aureus* ATCC430, *Salmonella enterica* ATCC14028, and *Escherichia coli* CVCC195) were cultured in LB broth at 37 °C until reaching 1 × 10^8^ CFU/mL.

### 2.2. Evaluation of the Probiotic Potential of L. animalis LA-1

#### 2.2.1. Simulated Gastrointestinal Tolerance

Gastrointestinal tolerance was assessed as previously described [35]. Briefly, LA-1 (1 × 10^9^ CFU/mL) was sequentially incubated in artificial gastric juice (3 h) and intestinal juice (8 h) at 37 °C. Viable counts were determined at each stage. All assays were performed in triplicate.

#### 2.2.2. Surface Hydrophobicity

Surface hydrophobicity of LA-1 was determined according to the methods of Dianawati et al. [36] and Hossain et al. [37], with minor modifications. After two subcultures, the cell suspension was adjusted to 1 × 10^9^ CFU/mL. Then, 3 mL of the LA-1 was mixed with 1 mL each of chloroform, xylene, and ethyl acetate. The mixture was vortexed for 3 min and then incubated statically at 37 °C for 25 min. The absorbance of the aqueous phase (A_1_) was measured at 600 nm. Surface hydrophobicity (%) was calculated using the following formula:

Hydrophobicity (%) = (1 − A_1_/A_0_) × 100

where A_0_ is the absorbance of the bacterial suspension before extraction and A_1_ is the absorbance after extraction.

#### 2.2.3. Antibiotic Susceptibility

Antibiotic susceptibility of LA-1 was tested using the disk diffusion method as previously described [35]. After two subcultures, the bacterial suspension was adjusted to 1 × 10^9^ CFU/mL. A 100 μL aliquot was spread onto MRS agar plates, and antibiotic disks containing tetracycline (30 μg), clindamycin (2 μg), gentamicin (10 μg), erythromycin (15 μg), chloramphenicol (30 μg), and ampicillin (10 μg) were placed on the surface. Plates were incubated at 37 °C for 24 h, and inhibition zones were measured to assess antibiotic sensitivity.

#### 2.2.4. Hemolytic Activity

Hemolytic activity of LA-1 was assessed according to a previously described method [38], with slight modifications. After two subcultures, the bacterial suspension was adjusted to 1 × 10^9^ CFU/mL and streaked onto blood agar plates supplemented with 5% (*w*/*v*) sterile sheep blood. Plates were incubated at 37 °C for 72 h. Hemolytic activity was evaluated by observing the type of hemolysis around the colonies. *Escherichia coli* CVCC195 was used as a positive control.

#### 2.2.5. Antibacterial Activity

Antibacterial activity of LA-1 was tested using the Oxford cup method as previously described [35]. Suspensions of *S. aureus* ATCC 430, *S. enterica* ATCC 14028, and *E. coli* CVCC195 (50 μL, 1 × 10^8^ CFU/mL) were spread on LB agar plates (2% *w*/*v* agar). LA-1 was adjusted to 1 × 10^9^ CFU/mL, and 100 μL was added to 6 mm Oxford cups placed on the plates. After incubation at 37 °C for 24 h, inhibition zones were measured.

#### 2.2.6. Cholesterol-Reducing Rate

The cholesterol-lowering activity of LA-1 was determined using the *O*-phthalaldehyde method [35]. A mixed acid solution (H_2_SO_4_–glacial acetic acid = 1:1, *v*/*v*) was prepared. LA-1 (1 × 10^9^ CFU/mL, 2% inoculum) was cultured in MRS-cholesterol medium (0.1 g/L cholesterol, 0.2 g/L bile salts) at 37 °C for 48 h. The control group contained no LA-1. After incubation, cultures were centrifuged, treated with ethanol, and centrifuged again. The supernatant was reacted with *O*-phthalaldehyde and mixed acid, and absorbance was measured at 550 nm. A standard curve was used for quantification. Cholesterol reduction rate (%) was calculated as follows:

Cholesterol reduction rate (%) = (1 − Cs/Cc) × 100

where Cs and Cc are the cholesterol contents (μg) in the sample and control, respectively. All tests were performed in triplicate.

#### 2.2.7. The Activity of Bile Salt Hydrolase (BSH)

Bile salt hydrolase (BSH) activity of LA-1 was assessed using the plate assay method as previously described [39]. LA-1, subcultured twice and adjusted to 1 × 10^9^ CFU/mL, was applied (20 μL) onto sterile paper discs placed on BSH agar plates. The plates were incubated anaerobically at 37 °C for 72 h. BSH activity was indicated by the formation of opaque precipitates around the discs.

#### 2.2.8. The Inhibition Activity of α-Glucosidase

LA-1 was subcultured twice, adjusted to 1 × 10^9^ CFU/mL, and inoculated into 50 mL MRS broth at 2% (*v*/*v*). Cultures were incubated at 37 °C for 24 h, then centrifuged at 8000× *g* for 5 min to collect the supernatant. The α-glucosidase inhibitory activity was evaluated according to the method of Apostolidis et al. [40], with slight modifications. Briefly, 50 μL of cell-free supernatant was mixed with 100 μL of α-glucosidase solution (1.0 U/mL) in a 96-well plate and pre-incubated at 25 °C for 10 min. Then, 50 μL of 5 mM *p*-nitrophenyl-α-d-glucopyranoside (pNPG) solution was added to initiate the reaction. After a further incubation at 25 °C for 5 min, absorbance at 405 nm was recorded before and after the reaction. A control was prepared using 50 μL of 0.1 M PBS instead of the sample. α-Glucosidase inhibition (%) was calculated using the following formula:

α-Glucosidase inhibition (%) = (1 − Cs/Cc) × 100

where Cs and Cc represent enzyme activity in the sample and control, respectively.

#### 2.2.9. Fatty Acid Absorption

Fatty acid absorption was evaluated according to a previously described method [41], with minor modifications. LA-1, subcultured twice and adjusted to 1 × 10^9^ CFU/mL, was inoculated at 2% (*v*/*v*) into MRS broth containing 0.5% (*w*/*v*) Brij58 and 0.25 mmol/L sodium palmitate. Cultures were incubated at 37 °C for 24 h. The residual fatty acid content in the medium was measured using a free fatty acid assay kit, which was purchased from Wuhan Shengzhiyuan Biotechnology Co., Ltd. (Wuhan, China).

### 2.3. Animals Experiment Design

Thirty-two SPF-grade male C57BL/6J mice (22 ± 2 g) were obtained from Vital River Laboratory Animal Technology Co., Ltd. (Beijing, China). Mice were housed under standard laboratory conditions (22 ± 2 °C, 55 ± 5% relative humidity, 12:12 h light-dark cycle) with free access to food and water. After a one-week acclimatization period, mice were randomly divided into four groups (*n* = 8 per group) based on body weight: control group (CT) and high-fat diet group (HFD, *n* = 24), which was further divided into three subgroups—HFD model group (HFD), positive control group (PC), and LA-1 treatment group (LA-1). The CT group received a standard chow diet, while the HFD group was fed a high-fat diet for 16 weeks to induce obesity. The CT and HFD groups were gavaged daily with 0.2 mL of 0.9% NaCl every day. The PC group received 0.2 mL of atorvastatin calcium solution (3 mg/kg), and the LA-1 group received 0.2 mL of LA-1 suspension (1 × 10^10^ CFU/mL). A medium dose of 2 × 10^9^ CFU/day was selected for probiotic administration based on previous studies [42], and the atorvastatin dose was determined according to published protocols [43]. The intervention lasted 16 weeks. Body weight, food intake, and remaining feed were recorded weekly. All procedures were approved by the Animal Ethics Committee of the Feed Research Institute, Chinese Academy of Agricultural Sciences (CAAS) under permit number IFR-CAAS20240415.

### 2.4. Sample Collection

At the end of the experimental period, all mice were fasted for 16 h and then euthanized for sample collection. Blood, liver, epididymal white adipose tissues (WATs), spleen, kidneys, and cecal contents were collected. Blood samples were centrifuged at 3000× *g* for 10 min at 4 °C, and the resulting serum was stored at −80 °C for further analysis. Cecal contents were rapidly frozen in liquid nitrogen. The liver, WATs, spleen, and kidneys were immediately weighed and recorded. Portions of the liver and WATs were fixed in 4% neutral-buffered paraformaldehyde for histological analysis, while the remaining tissues were snap-frozen in liquid nitrogen. All samples were stored at −80 °C until further use.

### 2.5. Biochemical Assay of Serum and Liver Tissues

Liver tissues were cleaned with ice-cold 0.1 M PBS, and 0.1 g was homogenized in 0.9 mL PBS using a tissue homogenizer. The homogenate was centrifuged at 3000× *g* for 15 min at 4 °C, and the supernatant was collected for biochemical analysis. Serum levels of total cholesterol (TC), triglycerides (TGs), low-density lipoprotein cholesterol (LDL-C), glucose (GLU), alanine aminotransferase (ALT), aspartate aminotransferase (AST), and non-esterified fatty acids (NEFAs), as well as hepatic levels of TC, TG, high-density lipoprotein cholesterol (HDL-C), LDL-C, GLU, ALT, and AST, were measured using commercial kits (Wuhan Shenzhyuan Biotechnology Co., Ltd., Wuhan, China) according to the manufacturer’s instructions.

### 2.6. Histological Evaluation

#### 2.6.1. Oil Red O Staining of Liver Tissue

Liver tissues were fixed in 4% paraformaldehyde (PFA), dehydrated, embedded in paraffin, and sectioned at 4–5 μm thickness. Sections were stained with Oil Red O solution to assess lipid accumulation, differentiated with 60% isopropanol, and counterstained with hematoxylin. Slides were mounted with glycerol gelatin and observed under a light microscope equipped with an imaging system.

#### 2.6.2. Hematoxylin and Eosin (HE) Staining of Liver and Adipose Tissues

Liver and adipose tissues were fixed in 4% PFA, dehydrated, embedded in paraffin, and cut into 4–5 μm sections. After staining with hematoxylin and eosin, histological changes were observed under a light microscope at 400× magnification.

### 2.7. Gut Microbiota Analysis: 16S rRNA Gene Sequencing

A total of 20 cecal content samples were subjected to 16S rRNA gene sequencing using the Illumina NovaSeq 6000 platform (Beijing Biomarker Technologies Co., Ltd., Beijing, China). Genomic DNA was extracted with the TGuide S96 Magnetic Stool DNA Kit (Tiangen Biotech, Beijing, China) according to the manufacturer’s instructions. The V3–V4 region of the 16S rRNA gene was amplified using primers 338F and 806R. PCR products were purified using Agencourt AMPure XP beads (Beckman Coulter, Indianapolis, IN, USA), quantified with the Qubit dsDNA HS Assay Kit (Thermo Fisher Scientific, Waltham, MA, USA), and pooled in equimolar concentrations. Sequencing was performed on the Illumina NovaSeq 6000 platform (Illumina, San Diego, CA, USA). Raw data were deposited in the NCBI SRA database (accession number: SRP566521). Denoising, chimera removal, and ASV generation were performed using DADA2 [44] in QIIME 2 (v2020.6) [45]. The analyses were performed using BMKCloud (www.biocloud.net, accessed on 1 June 2025).

### 2.8. Determination of Short-Chain Fatty Acids (SCFAs)

According to our previously described method [35], short-chain fatty acids (SCFAs) in cecal contents were quantified using gas chromatography–mass spectrometry (GC-MS) to assess microbial metabolic activity. Briefly, samples were homogenized in distilled water, acidified with 50% H_2_SO_4_, and extracted with methyl tert-butyl ether containing an internal standard. After centrifugation and purification, the supernatant was analyzed using a Shimadzu GC2030-QP2020 NX system (Shimadzu Corporation, Kyoto, Japan) equipped with a high-polarity fused silica capillary column (HPFFAP). SCFAs were detected in Scan/SIM mode under electron impact ionization (−70 eV), with helium as the carrier gas. GC-MS conditions followed standard protocols.

### 2.9. Untargeted Metabolomics Analysis of Liver

Untargeted metabolomic profiling of liver tissues was performed using an UHPLC system (Vanquish, Thermo Fisher Scientific, Waltham, MA, USA) with a Waters ACQUITY UPLC BEH Amide (2.1 mm × 50 mm, 1.7 μm; Waters Corporation, Milford, MA, USA) coupled to Orbitrap Exploris 120 mass spectrometer (Orbitrap MS, Thermo Fisher Scientific, Waltham, MA, USA) in both positive and negative ion modes. Pooled QC samples were used to monitor system stability. Raw data were processed for feature extraction, RSD filtering, missing value imputation (half minimum), and normalization by total ion current (TIC). Metabolites were identified using an in-house MS/MS spectral library (>20,000 standards) with ≥98% annotation accuracy. After preprocessing, 35,618 features were retained, including 2064 annotated metabolites.

Multivariate (PCA, OPLS-DA) and univariate (ANOVA) analyses were conducted using SIMCA (v18.0.1). Metabolites meeting VIP > 1 and *p* < 0.05 were considered significantly altered. The KEGG database was used for pathway enrichment and topological impact analysis.

### 2.10. Statistical Analysis

Data are expressed as mean ± standard deviation (SD). For two-group comparisons, Student’s *t*-test was used; for multiple-group comparisons, one-way ANOVA with Tukey’s post hoc test was applied. Normality and homogeneity of variance were assessed prior to parametric testing. Statistical significance was set at *p* < 0.05.

## 3. Results

### 3.1. Probiotic Properties of L. animalis LA-1 In Vitro

#### 3.1.1. Simulated Gastrointestinal Tolerance

**Figure 1:**
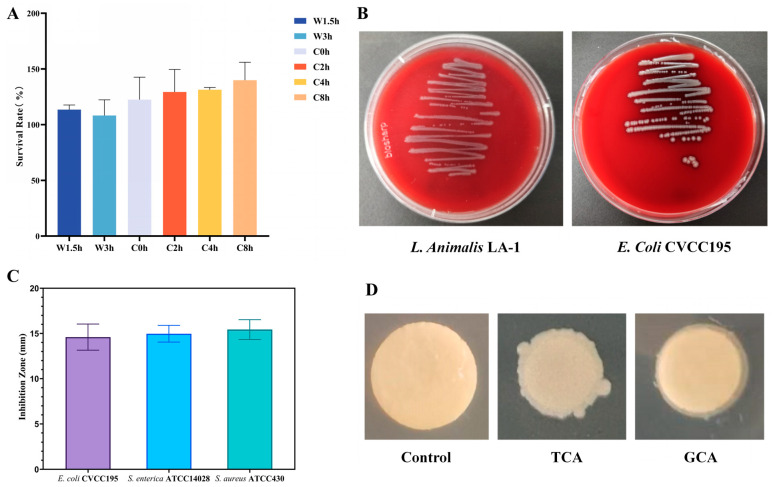
Probiotic properties of *L. animalis* LA-1 in vitro. (**A**) The tolerance of LA-1 in artificial gastrointestinal fluid. (**B**) Detection result of hemolytic test. (**C**) The antibacterial activity of LA-1. (**D**) Visual representation of the BSH activity of LA-1.

Probiotics must possess the ability to resist the destructive effects of gastric and intestinal fluids to survive and proliferate in the gastrointestinal tract. The survival ability of LA-1 was evaluated by assessing the number of viable cells in simulated gastric and intestinal fluids. The results showed that LA-1 had a survival rate of 108.25% in simulated gastric juice and 139.93% in simulated intestinal juice (Figure 1A), indicating that LA-1 not only survived but also partially proliferated under gastrointestinal conditions.

#### 3.1.2. Surface Hydrophobicity

Surface hydrophobicity is commonly used to evaluate bacterial adhesion ability [46]. LA-1 showed a high hydrophobicity of 75.86%, indicating strong surface adhesion potential.

#### 3.1.3. Antibiotic Susceptibility

The antibiotic susceptibility of LA-1 was evaluated, and the results showed that it was sensitive to all tested antibiotics, including tetracycline, clindamycin, gentamicin, erythromycin, chloramphenicol, ampicillin, and penicillin.

#### 3.1.4. Hemolytic Activity

Hemolytic activity is a key indicator for evaluating the safety of probiotic strains. As shown in Figure 1B, the positive control *E. coli* CVCC195 exhibited β-hemolysis, forming clear hemolytic zones (2–4 mm) around the colonies. In contrast, LA-1 showed γ-hemolytic activity, characterized by the absence of hemolysis, indicating that this strain is non-hemolytic and thus considered safe for probiotic use.

#### 3.1.5. Antibacterial Activity

The antagonistic activity of probiotic strains against pathogenic microorganisms is essential for maintaining host health. The antibacterial ability of LA-1 was evaluated against *S. aureus* ATCC430, *S. enterica* ATCC14028, and *E. coli* CVCC195 by measuring the diameter of inhibition zones. As shown in Figure 1C, LA-1 exhibited an inhibition zone of 15.43 mm against the Gram-positive bacterium *S. aureus* ATCC430. For the Gram-negative strains *S. enterica* ATCC14028 and *E. coli* CVCC195, the inhibition zones were 14.97 mm and 14.59 mm, respectively. These results indicate that LA-1 possesses broad-spectrum antimicrobial activity, effectively inhibiting both Gram-positive and Gram-negative pathogens.

#### 3.1.6. Cholesterol-Reducing Rate

LA-1 exhibited a cholesterol reduction rate of 50.03%, indicating excellent cholesterol-lowering ability.

#### 3.1.7. The Activity of Bile Salt Hydrolase (BSH)

In this study, the effect of LA-1 on taurocholic acid sodium salt hydrate (TCA) and glycocholic acid sodium salt hydrate (GCA) was evaluated. BSH activity was indicated by the presence of precipitation around the colonies, a rough colony surface, or both. As shown in Figure 1D, LA-1 exhibited hydrolytic activity against both TCA and GCA, demonstrating its BSH activity.

#### 3.1.8. The Inhibition Activity of α-Glucosidase

The inhibitory effect of the fermentation supernatant of LA-1 on α-glucosidase activity was evaluated. The results showed that LA-1 was capable of inhibiting α-glucosidase activity effectively. Specifically, LA-1 exhibited an inhibition rate of 54.54%, indicating a strong inhibitory effect. This result is comparable to the 35–60% inhibition range reported for lactic acid bacteria by Oh et al. [47].

#### 3.1.9. Fatty Acid Absorption

The fatty acid uptake ability of LA-1 was evaluated by incubating the strain in MRS broth supplemented with 0.25 mmol/L sodium palmitate for 24 h. After incubation, the broth was analyzed to determine the residual fatty acid concentration. The results showed that the addition of LA-1 significantly reduced the total fatty acid content in the medium, with an uptake rate of 67.61%.

### 3.2. L. animalis LA-1 Alleviated HFD-Induced Obesity in Mice

#### 3.2.1. *L. animalis* LA-1 Reduced Body Weight Gain and Organ Enlargement in Obese Mice

**Figure 2:**
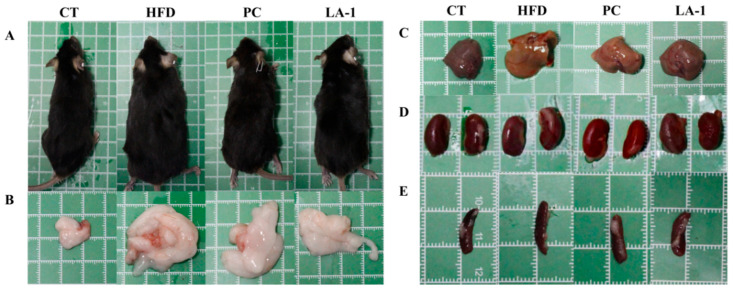
Effects of *L. animalis* LA-1 on the morphology of mice and their organs: (**A**) body; (**B**) epididymal WATs; (**C**) liver; (**D**) kidneys; (**E**) spleen.

As shown in Table 1, after 16 weeks of high-fat diet feeding, the average daily food intake in the HFD group was significantly lower than that in the control (CT) group (*p* < 0.05). However, no significant differences were observed among the HFD, PC, and LA-1 groups. Compared to the CT group, the HFD group exhibited a significant increase in body weight (*p* < 0.05) (Figure 2A). Notably, LA-1 intervention attenuated body weight gain relative to the HFD group, showing a stronger inhibitory effect than that of the PC group. These findings suggest that the reduction in body weight gain in the LA-1 group was not due to decreased food consumption.

Changes in body weight were also reflected in the relative weights of various organs and tissues. To assess the effect of LA-1 on fat accumulation in obese mice, organ indices of the liver, epididymal WATs, kidneys, and spleen were measured (Table 1 and Figure 2B–E). Compared with the CT group, the HFD group showed significantly increased liver, WAT, kidney, and spleen indices (*p* < 0.05). In contrast, the PC group showed significantly reduced liver, WAT, and spleen indices (*p* < 0.05), while the LA-1 group exhibited significant reductions in liver, WAT, kidney, and spleen indices (*p* < 0.05) compared to the HFD group.

#### 3.2.2. *L. animalis* LA-1 Prevented Lipid Accumulation in Serum and Liver of Obese Mice

Obesity is essentially a disorder of lipid metabolism, characterized by abnormal lipid levels in the blood, liver, and other tissues. As shown in Table 2, compared with the CT group, the HFD group showed significantly increased serum levels of TC, TG, LDL-C, GLU, ALT, and NEFA (*p* < 0.05). Intervention with LA-1 significantly reduced the levels of TG, GLU, ALT, AST, and NEFA compared with the HFD group (*p* < 0.05) and also lowered TC and LDL-C levels. The regulatory effects of LA-1 were superior to those observed in the PC group.

Similarly, as shown in Table 3, the hepatic levels of TC, TG, LDL-C, ALT, and AST were significantly increased in the HFD group compared with the CT group (*p* < 0.05), accompanied by elevated GLU levels and reduced HDL-C levels. Following intervention with LA-1, the levels of TC, TG, LDL-C, ALT, and AST were significantly reduced compared with the HFD group (*p* < 0.05), GLU levels were decreased, and HDL-C levels were increased. The effects observed in the LA-1 group were superior to those in the PC group.

#### 3.2.3. *L. animalis* LA-1 Prevented Liver and WAT Injury in Obese Mice

**Figure 3:**
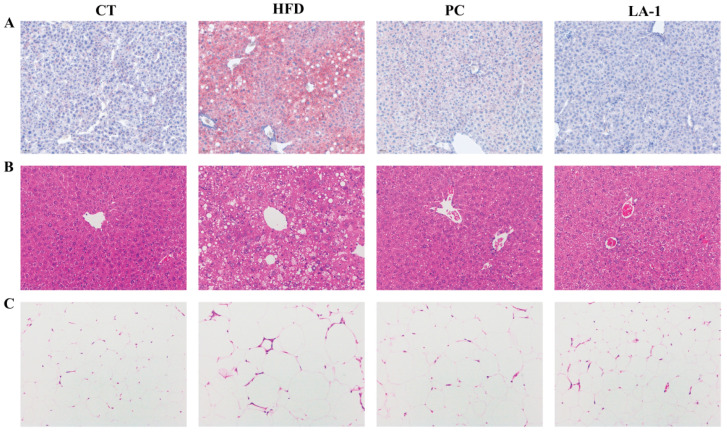
Intervention with *L. animalis* LA-1 reduced hepatic and epididymal fat accumulation in obese mice. (**A**) Oil Red O staining of liver tissue. (**B**) HE staining of liver tissue. (**C**) HE staining of epididymal WATs. Scale bar = 100 μm.

Oil Red O staining of liver tissues was performed to evaluate hepatic lipid accumulation. As shown in Figure 3A, the HFD group exhibited extensive lipid droplet deposition throughout the hepatic parenchyma, indicating severe steatosis. In contrast, the LA-1 group showed markedly reduced lipid accumulation, with hepatic morphology closely resembling that of the CT group. These results suggest that LA-1 has the potential to ameliorate hepatic dyslipidemia in obese mice.

Subsequently, H&E staining was conducted to assess histopathological changes in the liver and epididymal WATs. As shown in Figure 3B, liver sections from HFD-fed mice demonstrated disrupted lobular architecture, pronounced cellular ballooning, and widespread vacuolization, which were hallmarks of hepatic steatosis and tissue damage. In comparison, LA-1 treatment notably preserved hepatic structure, reduced vacuole formation, and improved overall histological integrity.

Similarly, Figure 3C shows that epididymal adipocytes in the HFD group were markedly enlarged, consistent with adipocyte hypertrophy induced by excessive energy intake. LA-1 intervention effectively alleviated this hypertrophy, with adipocytes appearing smaller and more uniform in size.

These results indicate that LA-1 treatment helps reduce hepatic injury and steatosis induced by a high-fat diet.

#### 3.2.4. *L. animalis* LA-1 Modulated the Structure and Composition of the Gut Microbiota in Obese Mice

**Figure 4:**
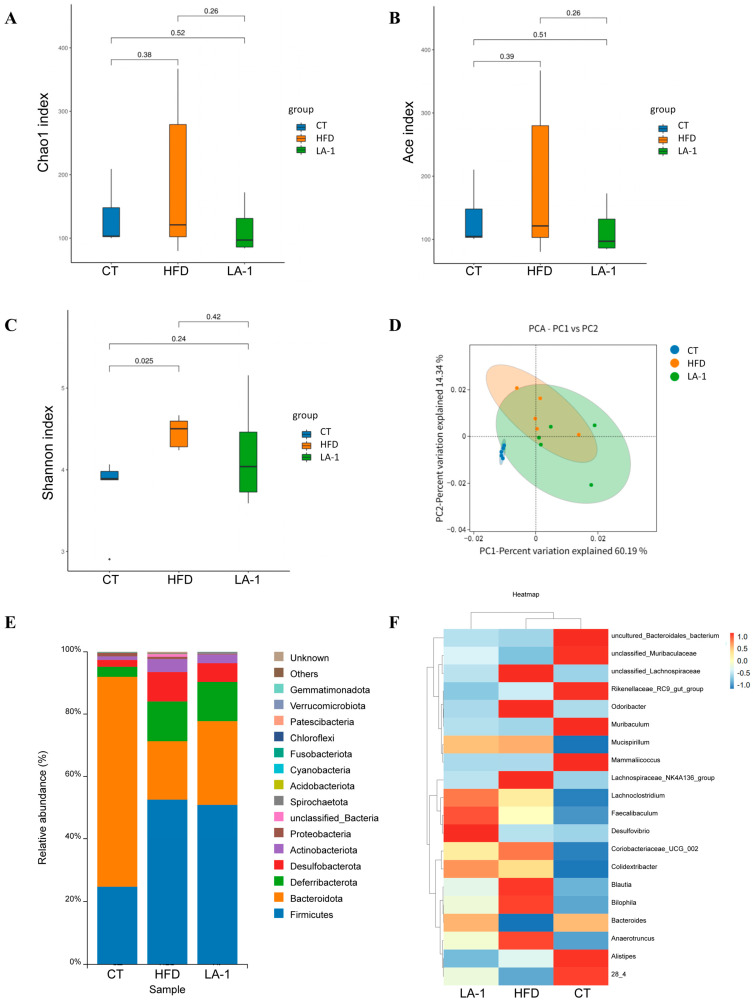
Effects of *L. animalis* LA-1 on gut microbiota composition in mice. (**A**) Chao1 index. (**B**) ACE index. (**C**) Shannon index. (**D**) PCA of gut microbiota. (**E**) Relative abundance of gut microbiota at the phylum level. (**F**) Heatmap of hierarchical clustering of bacterial genera.

To investigate the effects of LA-1 on the gut microbiota of HFD mice, 16S rRNA gene sequencing was performed to evaluate the microbial community composition in the cecal contents from the CT, HFD, and LA-1 groups. Bacterial diversity was assessed using the Chao1, ACE, and Shannon indices. As shown in Figure 4A–C, there was a significant difference in the Shannon index between the CT and HFD groups (*p* < 0.05), indicating that a high-fat diet markedly altered species diversity in the gut microbiota. Although the Shannon index in the LA-1 group did not differ significantly from that of the HFD group (*p* > 0.05), it tended to shift closer to the level observed in normal (CT) mice. No significant differences were observed in the Chao1 and ACE indices among the groups (*p* > 0.05), suggesting that species richness was comparable across all groups. To further assess compositional changes in the gut microbiota among the groups, principal component analysis (PCA) was performed. As shown in Figure 4D, the HFD group was completely separated from the CT group, highlighting the substantial impact of a high-fat diet on gut microbial composition. Following LA-1 treatment, the microbial clustering pattern shifted closer to that of the CT group, suggesting a partial restoration of gut microbiota structure toward a normal state.

A detailed analysis of bacterial composition at different taxonomic levels was conducted across the three groups. At the phylum level (Figure 4E), compared with the CT group, the HFD group exhibited a marked increase in the relative abundance of Firmicutes and a decrease in Bacteroidetes, indicating that a high-fat diet disrupts gut microbial balance in mice. Following LA-1 treatment, the relative abundance of Bacteroidetes increased compared to the HFD group (Table 4). Additionally, the Firmicutes/Bacteroidetes ratio was elevated in the HFD group relative to the CT group, while this ratio (F/B ratio) was reduced in the LA-1 group, suggesting that LA-1 alleviated high-fat diet-induced gut microbiota dysbiosis. Subsequently, bacterial composition at the genus level was analyzed (Figure 4F). Compared with the CT group, the HFD group showed increased abundances of *unclassified_Lachnospiraceae*, *Odoribacter*, *Mucispirillum*, *Lachnospiraceae_NK4A136_group*, *Coriobacteriaceae_UCG_002*, *Blautia*, *Bilophila*, and *Anaerotruncus*, whereas LA-1 treatment reduced the levels of these genera in HFD-fed mice. Meanwhile, the relative abundances of *unclassified_Muribaculaceae*, *Muribaculum*, *Bacteroides*, and *Lachnospiraceae bacterium 28_4* were decreased in the HFD group compared to the CT group, but this trend was reversed by LA-1 intervention.

These results demonstrate that LA-1 effectively attenuates gut microbiota dysbiosis caused by a high-fat diet.

#### 3.2.5. *L. animalis* LA-1 Promoted SCFAs Accumulation in the Cecal Contents of Obese Mice

SCFAs are known to play a critical role in lipid metabolism and the development of obesity [17]. To determine whether alterations in the gut microbiota led to changes in SCFA levels, SCFAs in the cecal contents were analyzed. The measured SCFAs included total SCFAs, acetic acid, propionic acid, butyric acid, isobutyric acid, pentanoic acid, isovaleric acid, and hexanoic acid. As shown in Table 5, the concentration of total SCFAs in the CT group was 1.7942 μg/mg, which significantly decreased to 1.0282 μg/mg in the HFD group (*p* < 0.001). After LA-1 intervention, the level increased to 1.1157 μg/mg. Compared with the CT group, the HFD group displayed significantly lower levels of total SCFAs, acetic acid, propionic acid, and butyric acid (*p* < 0.05), as well as reductions in isobutyric acid, pentanoic acid, isovaleric acid, and hexanoic acid. Notably, LA-1 treatment significantly increased total SCFAs in comparison to the HFD group (*p* < 0.001), including elevated concentrations of acetic acid, propionic acid, butyric acid, isobutyric acid, pentanoic acid, isovaleric acid, and hexanoic acid.

#### 3.2.6. *L. animalis* LA-1 Improved Hepatic Lipid and Amino Acid Metabolism in Obese Mice

**Figure 5:**
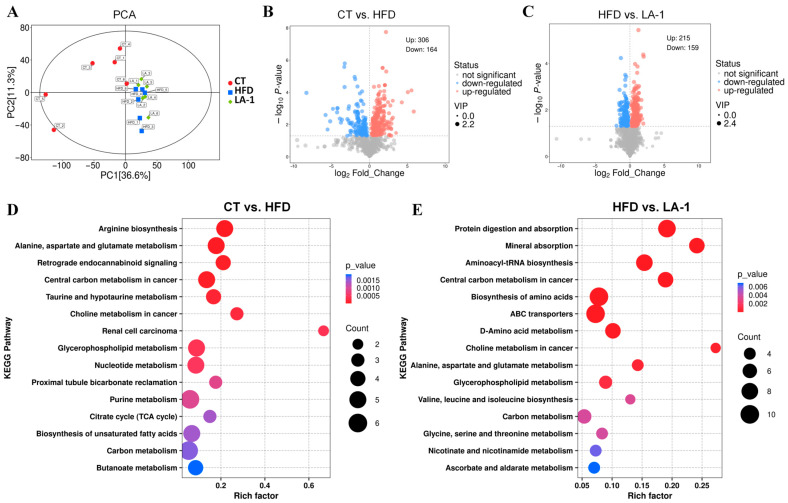
Effects of *L. animalis* LA-1 on hepatic metabolomics in mice. (**A**) PCA. (**B**) Volcano plot (CT vs. HFD). (**C**) Volcano plot (HFD vs. LA-1). (**D**) KEGG pathway enrichment analysis (CT vs. HFD). (**E**) KEGG pathway enrichment analysis (HFD vs. LA-1).

The liver plays a central role in lipid metabolism. Therefore, non-targeted metabolomic analysis was performed on liver samples from mice. PCA revealed distinct clustering patterns of metabolites among the CT, HFD, and LA-1 groups (Figure 5A). Both the HFD and LA-1 groups were separated from the CT group, indicating that a high-fat diet significantly altered hepatic metabolism in mice. Pairwise comparisons were performed among the three groups to identify metabolites with significant differences. The results of differential metabolite screening were visualized using volcano plots to intuitively display the overall distribution of metabolic changes between groups. Based on the criteria of *p* < 0.05 and VIP > 1, a total of 470 significantly altered metabolites were identified in the CT vs. HFD comparison, including 306 upregulated and 164 downregulated metabolites (Figure 5B). In the HFD vs. LA-1 comparison, 374 significantly altered metabolites were identified, of which 215 were upregulated and 159 were downregulated (Figure 5C).

**Figure 6:**
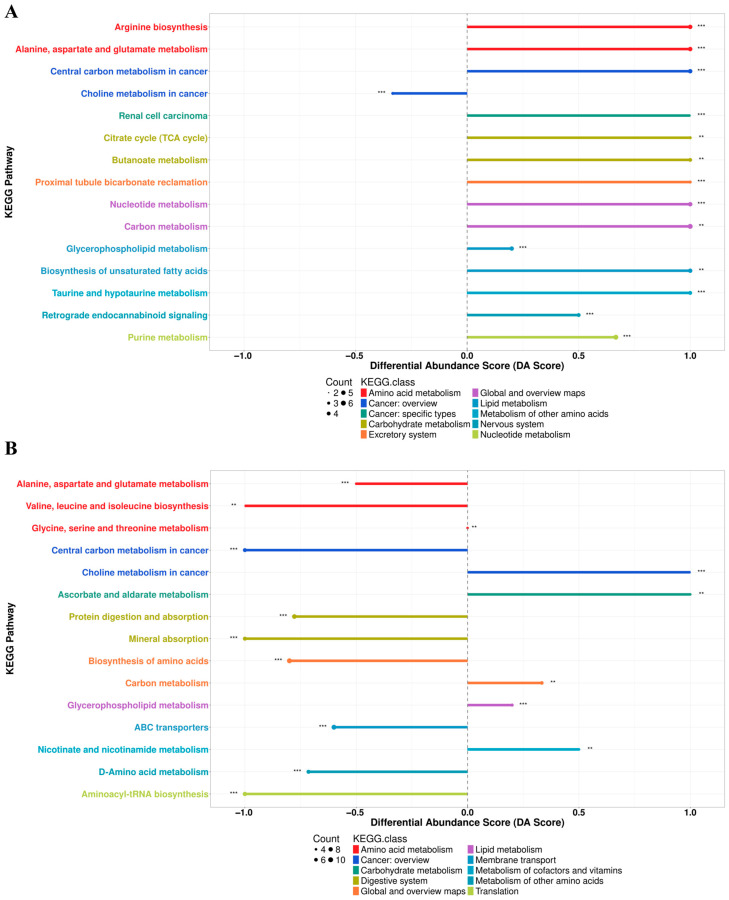
DA Score plots of KEGG pathway. (**A**) CT vs. HFD. (**B**) HFD vs. LA-1. The symbols **, and *** indicate significant differences at the *p* < 0.01, and *p* < 0.001 levels, respectively.

KEGG pathway enrichment analysis was performed for the differential metabolites identified in the CT vs. HFD and HFD vs. LA-1 comparisons (Figure 5D,E). The Differential Abundance Score (DA Score) plots revealed distinct metabolic trends (top 10 pathways). Compared with the CT group (Figure 6A), the HFD group showed an overall upregulation of metabolites involved in “arginine biosynthesis”, “alanine, aspartate and glutamate metabolism”, “central carbon metabolism in cancer”, “citrate cycle (TCA cycle)”, “butanoate metabolism”, “carbon metabolism”, “glycerophospholipid metabolism”, and “biosynthesis of unsaturated fatty acids”. In contrast, “choline metabolism in cancer” was downregulated. Conversely, when compared with the HFD group, pathways such as “alanine, aspartate and glutamate metabolism”, “valine, leucine and isoleucine biosynthesis”, “central carbon metabolism in cancer”, “biosynthesis of amino acids”, and “D-amino acid metabolism” were downregulated in LA-1 group (Figure 6B). Meanwhile, “choline metabolism in cancer”, “glycerophospholipid metabolism”, and “carbon metabolism” were upregulated.

**Figure 7:**
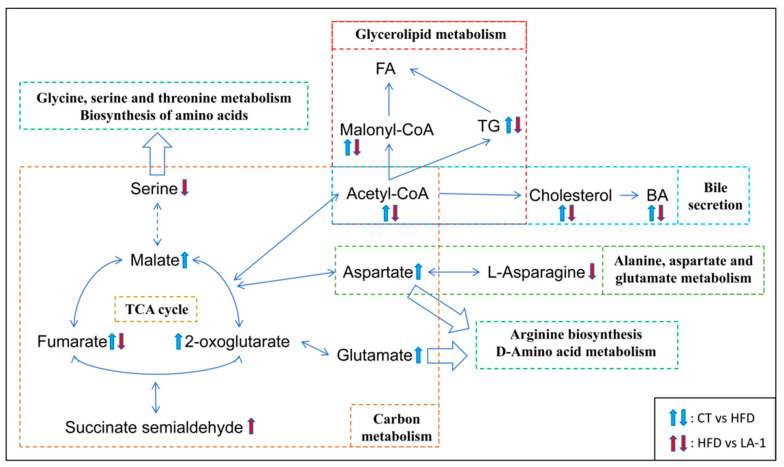
Pathway map illustrating the effects of *L. animalis* LA-1 on hepatic metabolism. Blue arrows represent changes between CT and HFD, while red arrows indicate changes between HFD and LA-1. Upward arrows indicate upregulation, and downward arrows indicate downregulation.

Specifically, as shown in Figure 7, we found that HFD increased the levels of Acetyl-CoA and several intermediates of the TCA cycle, including fumarate, malate, and 2-oxoglutarate. Additionally, levels of malonyl-CoA and TG, which are involved in glycerolipid metabolism, were elevated, along with increased levels of cholesterol and bile acids (BA). Moreover, HFD also elevated the levels of the amino acids aspartate and glutamate. After LA-1 treatment, the level of fumarate in the TCA cycle was reduced, while the level of succinate semialdehyde was increased. In amino acid metabolism, the levels of serine and L-asparagine were decreased. In addition, the levels of Acetyl-CoA, malonyl-CoA, TG, cholesterol, and BA were also reduced, collectively indicating a lipid-lowering effect.

These results suggest that LA-1 can alleviate hepatic lipid accumulation and metabolic imbalance by modulating key metabolites involved in energy, lipid, and amino acid metabolism, ultimately contributing to the mitigation of obesity in mice.

## 4. Discussion

In recent years, probiotics have garnered increasing attention for their potential to regulate gut microbiota and alleviate metabolic disorders. Among them, lactic acid bacteria have become a research focus due to their favorable safety profile and diverse biological functions [48,49]. In this study, we isolated a multifunctional probiotic strain, *L. animalis* LA-1, from the feces of healthy dogs and systematically evaluated its anti-obesity potential in an HFD-induced obese mouse model. Our findings provide theoretical rationale and experimental evidence for the development and application of LA-1 as a functional probiotic.

Probiotics play a critical role in maintaining gut health and intervening in metabolic diseases [19], and their in vitro functional characteristics are essential criteria for strain selection and application. In this study, LA-1 exhibited multiple typical probiotic properties in vitro, indicating strong application potential. Firstly, LA-1 demonstrated strong survival and colonization capacity. It exhibited high tolerance to simulated gastric and intestinal fluids, suggesting its ability to withstand the gastrointestinal environment and reach target sites. Additionally, its high surface hydrophobicity implies strong adhesion potential to intestinal epithelial surfaces, which may facilitate colonization and long-term host interaction [46]. In terms of safety, considering that antibiotic misuse and the resulting antimicrobial resistance have become major global public health concerns [50,51], it is crucial to identify strains sensitive to antibiotics. LA-1 was found to be susceptible to a range of commonly used clinical antibiotics, meeting basic safety requirements for probiotic industrial application. Moreover, the absence of hemolytic activity further supports its safety as a probiotic candidate. LA-1 also displayed broad-spectrum antimicrobial activity, effectively inhibiting the growth of several common pathogenic bacteria, including *S. aureus*, *S. enterica*, and *E. coli*, which may contribute to maintaining gut microbial homeostasis and preventing infections caused by pathogenic microorganisms.

Notably, LA-1 also exhibited significant potential in modulating lipid and glucose metabolism. LA-1 demonstrated a marked cholesterol-lowering effect, which may be attributed to mechanisms such as co-precipitation [52], cholesterol binding, or assimilation [53], suggesting its potential to reduce intestinal cholesterol levels. Meanwhile, LA-1 showed pronounced BSH activity, which catalyzes the deconjugation of bile salts into amino acids and free bile acids [54]. The latter can form insoluble complexes with cholesterol, thereby promoting cholesterol metabolism and excretion, ultimately contributing to reduced serum cholesterol levels [55]. In addition, LA-1 exhibited the ability to adsorb free fatty acids, indicating a potential to reduce lipid absorption within the intestinal lumen, thus mitigating fat accumulation. Regarding glycemic regulation, LA-1 effectively inhibited the activity of α-glucosidase, the key enzyme responsible for breaking down oligosaccharides into glucose during carbohydrate digestion [56]. Inhibition of this enzyme can delay glucose release and absorption, thereby lowering postprandial blood glucose levels [57] and reducing the conversion of glucose into fatty acids, which in turn suppresses triglyceride synthesis and adipocyte accumulation [58]. This mechanism not only aids in controlling blood glucose fluctuations but may also contribute synergistically to the alleviation of lipid metabolic disorders. Taken together, LA-1 possesses strong antimicrobial activity, an excellent safety profile, and multi-target regulatory effects on lipid and glucose metabolism, providing a solid foundation for its in vivo anti-obesity and metabolic regulatory functions.

Building on the in vitro functional validation, we further evaluated the in vivo anti-obesity effects of LA-1 using an HFD-induced obese mouse model. The results demonstrated that LA-1 supplementation significantly alleviated HFD-induced obesity-related phenotypic changes. Compared to the HFD model group, mice in the LA-1 treatment group exhibited a markedly slower rate of body weight gain and a downward trend in final body weight, suggesting that LA-1 may suppress weight gain by modulating energy metabolism or inhibiting fat accumulation. Consistent with the changes in body weight, the indices of major organs (including the liver, spleen, and kidneys) and WATs in the LA-1 group were lower than those in the model group, indicating a potential effect in reducing fat deposition and mitigating organ enlargement. As the central organ of lipid metabolism, the liver is prone to lipid accumulation and steatosis under obese conditions [59]. Histological analysis revealed that LA-1 improved hepatic tissue integrity, attenuated hepatic steatosis, and suppressed adipocyte hypertrophy, further confirming its ability to reduce liver fat accumulation. Moreover, LA-1 supplementation resulted in reduced levels of total TC, TG, LDL-C, and GLU in both serum and liver tissues, while the HDL-C level showed an increasing trend. These findings indicate that LA-1 can improve lipid profiles and alleviate hepatic lipid accumulation. Notably, the in vivo lipid-lowering effects are consistent with the cholesterol-binding capacity and BSH activity observed in vitro, suggesting that LA-1 may exert its effects through modulation of cholesterol metabolism pathways.

The gut microbiota serves as a critical regulator of host metabolic homeostasis, and its dysbiosis is closely associated with obesity and various metabolic disorders [60]. HFD feeding has been shown to significantly disrupt gut microbial ecology, leading to a reduction in beneficial microbes and an enrichment of potentially pathogenic bacteria, thereby exacerbating lipid metabolic dysregulation and systemic inflammation [61,62]. In this study, 16S rRNA gene sequencing revealed that LA-1 supplementation partially reversed HFD-induced gut microbiota dysbiosis, demonstrating a pronounced capacity to modulate the intestinal microbial ecosystem. HFD markedly altered the gut microbial structure, characterized by an increased relative abundance of *Firmicutes* and a significant decrease in *Bacteroidetes*, resulting in an elevated F/B ratio, a well-recognized indicator of obesity-associated microbial imbalance [63]. LA-1 intervention effectively reduced the F/B ratio, indicating its potential to correct HFD-induced microbial shifts. Further analysis revealed that LA-1 promoted the recovery or enrichment of several beneficial bacterial genera in HFD-fed mice, such as *Bacteroides* and *Muribaculaceae*. *Bacteroides* are major producers of SCFAs, particularly acetate and propionate, and their increased abundance may contribute to the improvement of the intestinal metabolic environment [64]. *Muribaculaceae*, a common group of anaerobes in the murine gut, possesses core metabolic capabilities for degrading complex polysaccharides and producing diverse SCFAs, with demonstrated anti-inflammatory and glycemic regulatory potential [65]. The enrichment of these taxa following LA-1 supplementation suggests a positive role in restoring microbial homeostasis. Conversely, LA-1 intervention significantly suppressed several microbial taxa associated with inflammation and metabolic disorders, including *unclassified_Lachnospiraceae*, *Mucispirillum*, *Coriobacteriaceae_UCG_002*, and *Bilophila*. Notably, the abundance of *unclassified_Lachnospiraceae* has been positively correlated with type 2 diabetes [66]. *Mucispirillum* is considered an opportunistic pathogen linked to colitis, autoimmunity, and tumorigenesis [67]. HFD has been shown to increase *Coriobacteriaceae* abundance, which may promote colorectal tumor development via the CPT1A–ERK signaling axis [68]. Additionally, *Bilophila* proliferation under HFD conditions is associated with inflammation, impaired glucose metabolism, and hepatic steatosis [69]. The suppression of these potentially pathogenic microbes by LA-1 may help attenuate intestinal inflammation, restore microbial balance, and ultimately contribute to the improvement of host lipid metabolism.

SCFAs are key metabolites produced by gut microbiota through the fermentation of dietary fibers. They play essential roles in maintaining intestinal barrier integrity, regulating energy metabolism, modulating inflammatory responses, and shaping host immunity [70,71,72]. To further elucidate the mechanism by which LA-1 influences host metabolism via the gut microbiota–metabolite axis, we quantitatively analyzed the major SCFAs in cecal contents. The results showed that an HFD significantly reduced the total SCFA concentration, particularly acetate, propionate, and butyrate levels. Notably, LA-1 supplementation effectively reversed this trend (*p* < 0.05). Among these, acetate—the most abundant SCFA—plays diverse roles in hepatic fatty acid synthesis and regulation of lipid oxidation [73,74]. Propionate can improve liver injury via the gut–liver axis, modulate appetite-regulating hormones, and regulate glucose metabolism, thereby reducing fat accumulation [75,76,77]. Butyrate, the primary energy source for colonic epithelial cells, is also crucial for maintaining epithelial barrier function and suppressing intestinal inflammation [78,79]. These findings suggest that LA-1 may improve host metabolic status through a multi-target mechanism involving the elevation of SCFA levels. The observed changes in SCFA concentrations closely correlated with the restoration of gut microbiota structure. Specifically, the increased abundance of SCFA-producing genera such as *Bacteroides* and *Muribaculaceae* in the LA-1 group likely contributed directly to enhanced SCFA synthesis. Moreover, as a lactic acid bacterium, LA-1 itself is also capable of SCFA production [80]. Importantly, the signaling activity of SCFAs in host metabolic regulation should not be overlooked. SCFAs can activate intestinal G protein-coupled receptors (e.g., GPR41 and GPR43), thereby modulating fatty acid oxidation, glucagon-like peptide-1 (GLP-1) secretion, and satiety signaling pathways, ultimately influencing systemic energy metabolism and insulin sensitivity [81,82,83,84]. These metabolite-mediated effects may serve as a key mechanism underlying the anti-obesity and metabolic regulatory properties of LA-1.

LA-1 may also exert immunomodulatory effects that contribute to the observed improvements in host physiology. Obesity and high-fat diet feeding are known to induce a low-grade chronic inflammatory state, characterized by elevated levels of pro-inflammatory cytokines, which in turn exacerbate insulin resistance, lipid accumulation, and hepatic injury [85]. Notably, the increased abundance of anti-inflammatory genera such as *Muribaculaceae* and *Bacteroides* may play a role in suppressing systemic inflammation [65,78]. In particular, these taxa are known producers of SCFAs, which can inhibit NF-κB signaling and promote the expansion of regulatory T cells via activation of G-protein-coupled receptors [86,87]. Such mechanisms have been shown to attenuate inflammatory cytokine expression in various metabolic disease models. These observations raise the possibility that LA-1 mitigates metabolic dysfunction not only through direct metabolic modulation but also by reducing inflammation via microbiota-mediated immune regulation. Future studies incorporating cytokine profiling and immune cell characterization will be essential to validate this hypothesis and further elucidate the immunometabolic mechanisms underlying LA-1’s therapeutic potential.

The liver is a central organ in regulating host energy metabolism and lipid biosynthesis, and its metabolic homeostasis is essential for combating obesity [88,89]. To further elucidate the mechanisms by which LA-1 modulates metabolic disturbances induced by an HFD, we conducted untargeted metabolomic profiling of hepatic tissues in mice. PCA analysis revealed distinct clustering patterns among the CT, HFD, and LA-1 groups, indicating that HFD induced significant perturbations in the hepatic metabolic network. KEGG pathway enrichment analysis showed that HFD markedly upregulated pathways involved in the TCA cycle, carbon metabolism, unsaturated fatty acid biosynthesis, and glycerophospholipid metabolism. Correspondingly, levels of key metabolites—such as acetyl-CoA, malonyl-CoA, TG, cholesterol, and bile acids—were significantly elevated, reflecting a state of excessive lipid synthesis and accumulation in the liver. Furthermore, several TCA cycle intermediates, including fumarate, malate, and 2-oxoglutarate, were elevated, suggesting enhanced aerobic metabolic activity likely due to excess energy intake. In terms of amino acid metabolism, HFD feeding significantly increased levels of glutamate and aspartate—amino acids that serve as metabolic nodes in carbon metabolism and provide precursors for fatty acid synthesis via the TCA cycle [90]. Strikingly, the LA-1 intervention substantially reversed these metabolic abnormalities. Fumarate levels in the TCA cycle were reduced, while succinate semialdehyde levels increased, indicating that LA-1 may alleviate metabolic overload by redirecting intermediate metabolic fluxes. Moreover, decreased levels of acetyl-CoA and malonyl-CoA, along with reductions in TG, cholesterol, and bile acids, suggest that LA-1 effectively inhibits hepatic lipid synthesis and storage, contributing to an overall lipid-lowering effect.

Importantly, acetyl-CoA is a central metabolic intermediate that links lipogenesis and the TCA cycle [91]. Elevated levels of acetyl-CoA under HFD conditions may reflect excessive influx of fatty acid-derived substrates into mitochondria and promote lipogenesis [91]. The observed reduction in acetyl-CoA following LA-1 intervention may indicate a restoration of energy balance, possibly through enhanced substrate utilization efficiency or reduced lipogenic drive, thus contributing to improved hepatic energy homeostasis. Significant alterations were also observed in amino acid metabolism. Metabolite levels of serine and L-asparagine were reduced following LA-1 treatment. Glutamate–aspartate metabolism and branched-chain amino acid (BCAA) biosynthesis were notably downregulated. Elevated BCAA levels are typically associated with obesity, insulin resistance, and type 2 diabetes [92]. Therefore, the observed reductions imply a rebalancing of amino acid fluxes, which may help alleviate the metabolic burden imposed by HFD-induced glucose and lipid dysregulation. Moreover, amino acids can serve as anaplerotic substrates for the TCA cycle by being converted into key intermediates such as 2-oxoglutarate, pyruvate, and fumarate. Under HFD conditions, the elevated levels of amino acids may thus fuel the TCA cycle, enhancing the production of acetyl-CoA. As a central precursor for de novo lipogenesis, excess acetyl-CoA contributes to hepatic lipid accumulation [93]. In this context, the observed reductions in amino acid levels, TCA cycle intermediates, and acetyl-CoA following LA-1 intervention suggest that LA-1 may suppress hepatic lipid synthesis by attenuating amino acid-driven TCA cycle flux and limiting acetyl-CoA availability. This metabolic rerouting may represent a key mechanism by which LA-1 alleviates lipid overload and restores hepatic metabolic balance.

At the pathway level, LA-1 modulated several key metabolic routes, including “choline metabolism in cancer”, “glycerophospholipid metabolism”, and “carbon metabolism”. Restoration of phospholipid metabolism may not only help maintain hepatocellular membrane integrity but also reduce lipid droplet formation, thereby lowering the risk of hepatic steatosis at its source [94,95]. In summary, LA-1 exerts a multi-pathway regulatory effect on hepatic metabolism, significantly ameliorating HFD-induced disturbances in carbon metabolism, amino acid metabolism, and lipid biosynthesis. This metabolic reprogramming likely underlies its ability to reduce hepatic lipid accumulation, alleviate metabolic stress, and restore systemic energy balance, providing a robust molecular basis for its anti-obesity effects.

While this study provides comprehensive insights into the anti-obesity effects of *L. animalis* LA-1 through multi-omics analyses, including gut microbiota profiling, SCFA quantification, and hepatic metabolomics, certain limitations should be noted. The mechanistic link between increased SCFA levels and host metabolic improvements remains indirect, as we did not assess downstream signaling pathways such as GPR41, GPR43, or histone deacetylase inhibition in relevant tissues. In addition, inflammatory cytokines associated with obesity-related immune responses, including TNF-α, IL-6, and IL-10, were not measured, limiting our understanding of the immunometabolic effects of LA-1. Although we observed significant shifts in gut microbial composition, particularly in the F/B ratio and specific genera, the causal relationship between these changes and metabolic outcomes was not functionally validated. Future studies incorporating transcriptomic, immunological, and receptor-level analyses are warranted to further elucidate the molecular pathways underlying the observed probiotic effects.

## 5. Conclusions

In this study, we identified a novel probiotic strain, *Ligilactobacillus animalis* LA-1, isolated from healthy canine feces. We systematically evaluated its anti-obesity potential using both in vitro assays and in vivo multi-omics analyses in a high-fat diet (HFD)-induced obese mouse model. In vitro, LA-1 showed strong tolerance to simulated gastric and intestinal fluids, high surface hydrophobicity, and broad antimicrobial activity. It was also non-hemolytic and sensitive to common antibiotics, confirming its safety. Moreover, LA-1 exhibited functional properties, including cholesterol-lowering activity, bile salt hydrolase (BSH) activity, α-glucosidase inhibition, and fatty acid absorption. In vivo, LA-1 supplementation significantly reduced body weight gain and lipid accumulation in both liver and serum. It also improved liver histology and attenuated white adipose tissue hypertrophy. Furthermore, LA-1 partially restored gut microbial diversity, increased SCFA production, and reprogrammed hepatic metabolism, as revealed by untargeted metabolomics. These findings suggest that LA-1 is a promising probiotic candidate for the prevention or treatment of obesity and metabolic disorders.

## Figures and Tables

**Table 1:** Effect of *L. animalis* LA-1 on growth performance in mice.

Items	CT	HFD	PC	LA-1	*p* Value
Food intake (g/week)	50.44 ± 0.37 a	36.50 ± 0.32 b	36.05 ± 0.09 b	36.64 ± 1.62 b	<0.001
Initial body weight (g/each)	22.11 ± 0.03 a	22.37 ± 0.02 a	22.15 ± 0.15 a	22.14 ± 0.56 a	0.788
Final body weight (g/each)	26.95 ± 0.21 b	34.61 ± 2.69 a	33.29 ± 0.08 a	31.93 ± 0.55 a	0.019
Body weight gain (g/each)	4.84 ± 0.18 b	12.23 ± 2.67 a	11.15 ± 0.08 a	9.79 ± 0.01 a	0.018
Liver index	3.44 ± 0.13 b	4.08 ± 0.04 a	3.48 ± 0.19 b	3.64 ± 0.17 b	0.019
WATs index	1.71 ± 0.12 c	6.45 ± 0.83 a	4.66 ± 1.02 b	3.91 ± 0.68 b	<0.001
Kidney index	1.30 ± 0.02 ab	1.43 ± 0.10 a	1.30 ± 0.08 ab	1.22 ± 0.05 b	0.023
Spleen index	0.25 ± 0.01 b	0.38 ± 0.11 a	0.25 ± 0.03 b	0.25 ± 0.03 b	0.014

^a–c^ Different lowercase letters mean significant differences (*p* < 0.05).

**Table 2:** Effect of *L. animalis* LA-1 on serum biochemical parameters in mice.

Items	CT	HFD	PC	LA-1	*p* Value
TC (mmol/L)	3.03 ± 0.14 b	5.03 ± 0.35 a	4.61 ± 0.29 a	4.70 ± 0.16 a	<0.001
TG (mmol/L)	1.15 ± 0.04 c	1.65 ± 0.09 a	1.37 ± 0.17 b	1.27 ± 0.10 bc	<0.001
LDL-C (mmol/L)	0.77 ± 0.07 b	1.32 ± 0.08 a	1.25 ± 0.05 a	1.26 ± 0.03 a	<0.001
GLU (mmol/L)	6.00 ± 0.58 c	11.18 ± 0.79 a	8.98 ± 1.26 b	6.10 ± 0.33 c	<0.001
ALT (U/L)	31.69 ± 1.07 b	42.21 ± 3.58 a	34.32 ± 2.28 b	22.70 ± 1.82 c	<0.001
AST (U/L)	128.77 ± 7.65 ab	165.44 ± 17.63 a	146.83 ± 3.82 b	116.15 ± 8.27 c	<0.001
NEFA (mmol/L)	2.40 ± 0.11 b	2.88 ± 0.15 a	2.41 ± 0.19 bc	2.64 ± 0.26 b	0.011

^a–c^ Different lowercase letters mean significant differences (*p* < 0.05).

**Table 3:** Effect of *L. animalis* LA-1 on hepatic biochemical parameters in mice.

Items	CT	HFD	PC	LA-1	*p* Value
TC (µmol/g)	43.22 ± 3.16 b	53.27 ± 1.97 a	39.54 ± 3.35 b	38.11 ± 1.27 b	<0.001
TG (µmol/g)	102.37 ± 12.59 c	204.84 ± 34.63 a	150.18 ± 14.13 b	132.95 ± 17.16 bc	<0.001
HDL-C (µmol/g)	8.83 ± 0.13 a	6.06 ± 1.20 b	9.76 ± 1.50 a	9.58 ± 0.46 a	0.029
LDL-C (µmol/g)	19.23 ± 1.07 c	29.25 ± 1.23 a	23.77 ± 1.83 b	17.66 ± 3.69 c	<0.001
GLU (mmol/g)	1.32 ± 0.17 a	1.83 ± 0.20 a	1.34 ± 0.29 a	1.49 ± 0.05 a	0.168
ALT (U/g)	770.53 ± 57.90 c	1265.76 ± 135.10 a	1034.99 ± 65.23 b	825.89 ± 94.91 c	<0.001
AST (U/g)	2368.14 ± 200.34 b	3463.37 ± 397.84 a	2754.15 ± 345.32 b	2385.84 ± 131.85 b	0.005

^a–c^ Different lowercase letters mean significant differences (*p* < 0.05).

**Table 4:** The abundance of Firmicutes and Bacteroidetes and the F/B ratio of mice.

Groups	CT	HFD	LA-1
Firmicutes	24.71%	52.54%	50.86%
Bacteroidetes	67.24%	18.77%	26.86%
F/B ratio	37%	280%	189%

**Table 5:** Effects of *L. animalis* LA-1 intervention on SCFA levels in the cecal contents of mice.

Items	CT	HFD	LA-1	*p* Value
Acetic acid (μg/mg)	0.8355 ± 0.086 a	0.501 ± 0.0.0506 b	0.5074 ± 0.0299 b	0.018
Propionic acid (μg/mg)	0.4446 ± 0.0078 a	0.1687 ± 0.0013 b	0.1801 ± 0.0366 b	0.002
Butyric acid (μg/mg)	0.4929 ± 0.0785 a	0.2600 ± 0.0037 b	0.2609 ± 0.0527 b	0.037
Isobutyric acid (μg/mg)	0.0367 ± 0.0021 a	0.0347 ± 0.0012 a	0.0484 ± 0.0100 a	0.187
Pentanoic acid (μg/mg)	0.0514 ± 0.0012 a	0.057 ± 0.025 a	0.0681 ± 0.0182 a	0.668
Isovaleric acid (μg/mg)	0.0320 ± 0.0019 a	0.0315 ± 0.0001 a	0.04632 ± 0.0087 a	0.100
Hexanoic acid (μg/mg)	0.0045 ± 0.0001 a	0.0024 ± 0.0000 a	0.0044 ± 0.0012 a	0.087
Total SCFAs concentration (μg/mg)	1.7942 ± 0.0217 a	1.0282 ± 0.0273 c	1.1157 ± 0.0213 b	<0.001

^a–c^ Different lowercase letters mean significant differences (*p* < 0.05).

## Data Availability

The original contributions presented in the study are included in the article; further inquiries can be directed at the corresponding authors.
